# Supplementary figures and images for: Granzyme B PET Imaging Enables Detection of CAR T-Cell Therapy Response in a Human Melanoma Mouse Model
Source: Diagnostics (Basel). 2025 Nov 30;15(23):3058. doi: 10.3390/diagnostics15233058 (PMC12692077; doi:10.3390/diagnostics15233058)

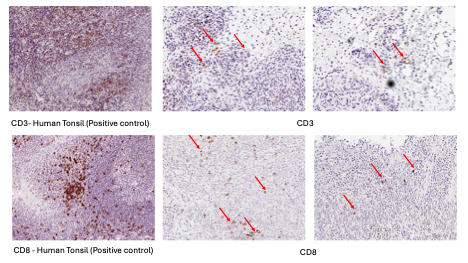

Supplement: Supplementary file 1 [file diagnostics-15-03058-s001.zip › Figure S1.png]

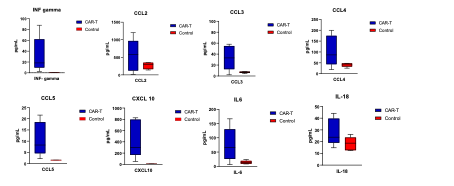

Supplement: Supplementary file 1 [file diagnostics-15-03058-s001.zip › Figure S2.png]
